# 

**Published:** 1987-02

**Authors:** 


					Editor
Mr M. G. Wilson
40 Coombe Lane, Bristol BS9 2BJ
Tel: 0272-682320
Assistant Editor
Dr P. R. Goddard
Editorial Committee
Mr B. P. Jones (Secretary),
Medical Librarian, Bristol University
Dr J. L. Burton
Correspondents
Dr A. E. Adam (Taunton)
Dr J. D. Davies
Dr J. Jancar .
Professor D. J. Pereira Gray (Exeter)
Dr H. G. Mather
Professor G. M. Stirratt
Mr C. Teasdale (Plymouth)
Dr J. L. G. Thomson
^r M. J. Whitfield
Professor G. K. Wilcock
Professor R. C. N. Williamson
Dr D. W. Wright
Bristol medico-chirurgical society
President
Mr H. B. Griffith
Secretary
Mr R. n. Baird,
23 Old Sneed Park, Bristol BS9 1RG
Treasurer
^r N. C. Tricks,
The Mill, Wrington, Bristol
Published by
Hermiston Publications,
BMA Scottish House,
7 Drumsheugh Gardens,
Edinburgh EH3 7QP
Production Manager
June Macnab
typesetting by
Avon Communications Ltd.,
Avon House, Nailsea, Bristol BS19 2UJ
Scottish County Press, Sherwood Industrial Estate,
Bonnyrigg, Midlothian
?Established 1883i
BRISTOL
MEDICO
CHIRIJRG1CAL
JOURNAL
FEBRUARY 1987
Volume 102 (i)
Published quarterly and distributed free of charge on
request to doctors in the South West of England.
Overseas subscription ?12 per annum.
What I know remains unknown, unless by me to others
shown.
Lucilius (180-102 bc)
^OTICE to contributors
ontributions are invited especially from West of England authors on a variety of subjects, e.g. Original articles relating to the practice
rnedicine, reports on the contemporary medical scene, obituaries, historical accounts, recollections of colleagues and events worthy
? be remembered and liable to be forgotten. An article of 2,000 words with 2 illustrations will occupy 2 pages and this is a suggested,
ough by no means an obligatory length. Articles are preferred in the form proposed by the International Committee of Medical
t?rs (Vancouver System)?pamphlet obtainable from Editor of BMJ., BMA. House, Tavistock Square, London, WC1H 9JR.
Bristol
medicine
Bristol Medicine acknowledges with gratitude the
support it receives from Pfizer Ltd.
IMPORTANT NOTICE
The Journal has been distributed free to all doctors in the West of England in 1986. If they
wish to continue receiving it gratis in 1987 will they please write to:
HERMISTON PUBLICATIONS, 7 Drumsheugh Gardens, Edinburgh, EH3 7QP.
Those who formerly received it as subscribers need not request it.
5

				

